# Oligonucleotide analogues with cationic backbone linkages

**DOI:** 10.3762/bjoc.14.111

**Published:** 2018-06-04

**Authors:** Melissa Meng, Christian Ducho

**Affiliations:** 1 Department of Pharmacy, Pharmaceutical and Medicinal Chemistry, Saarland University, Campus C2 3, 66123 Saarbrücken, Germany

**Keywords:** backbone modifications, cations, DNA, oligonucleotides, zwitterions

## Abstract

Their unique ability to selectively bind specific nucleic acid sequences makes oligonucleotides promising bioactive agents. However, modifications of the nucleic acid structure are an essential prerequisite for their application in vivo or even in cellulo. The oligoanionic backbone structure of oligonucleotides mainly hampers their ability to penetrate biological barriers such as cellular membranes. Hence, particular attention has been given to structural modifications of oligonucleotides which reduce their overall number of negative charges. One such approach is the site-specific replacement of the negatively charged phosphate diester linkage with alternative structural motifs which are positively charged at physiological pH, thus resulting in zwitterionic or even oligocationic backbone structures. This review provides a general overview of this concept and summarizes research on four according artificial backbone linkages: aminoalkylated phosphoramidates (and related systems), guanidinium groups, *S*-methylthiourea motifs, and nucleosyl amino acid (NAA)-derived modifications. The synthesis and properties of the corresponding oligonucleotide analogues are described.

## Introduction

**Figure 1:**
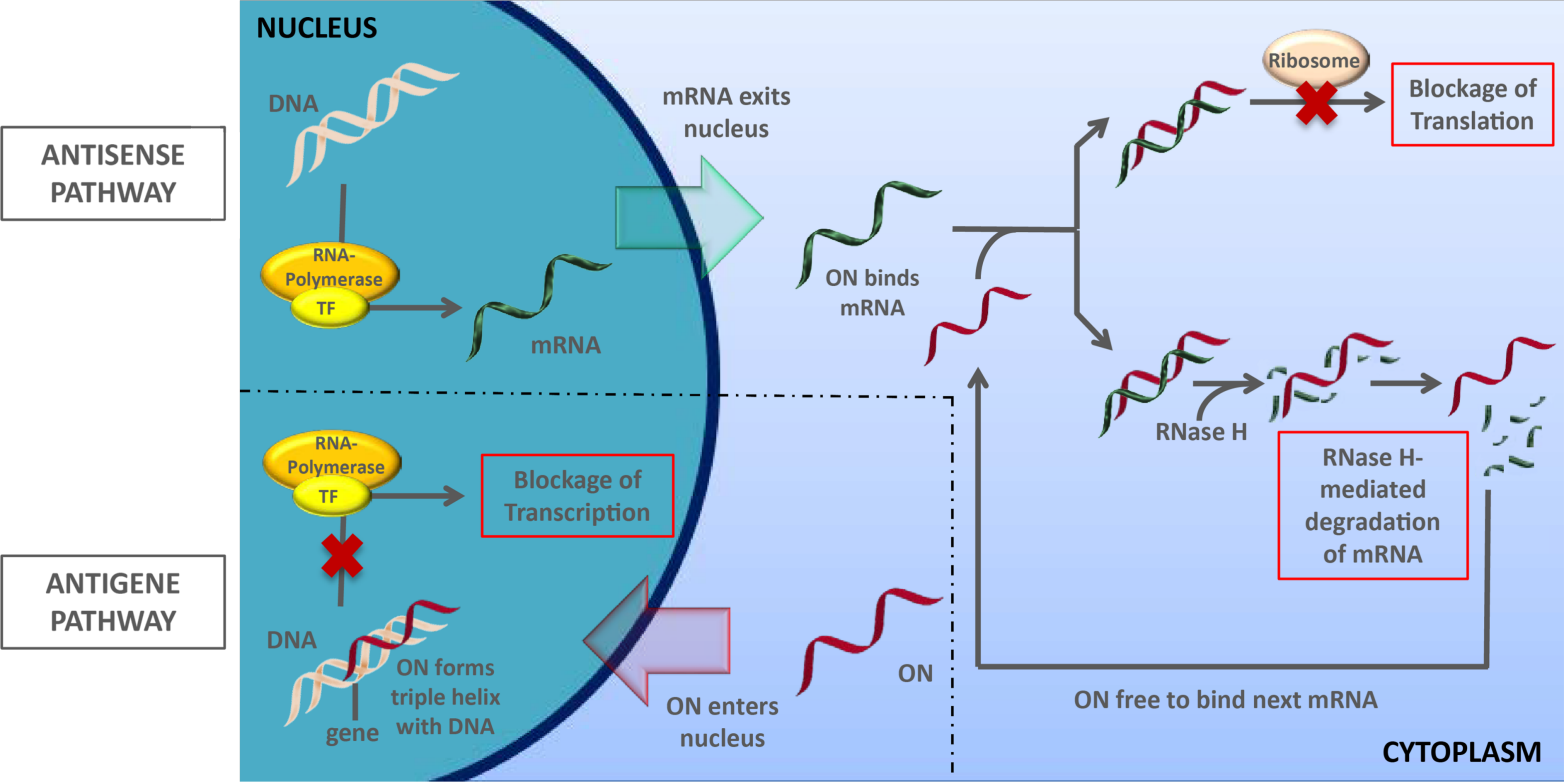
Biological action of single-stranded oligonucleotides (ON): antigene and antisense pathways.

Oligonucleotides have the unique ability to bind endogenous nucleic acids in a selective and sequence-specific manner. They can therefore modulate biological functions via different mechanisms [1]. Single-stranded oligonucleotides (ONs) can act in cellulo mainly via two different pathways (Figure 1). In the antigene pathway [2], the ON enters the nucleus and binds to double-stranded DNA to form a triple helix. The triple helix is not a substrate for the transcription machinery, and hence, RNA biosynthesis (and therefore protein formation) is blocked. In the antisense pathway [3], the ON binds to single-stranded mRNA in the cytoplasm, thus furnishing a duplex structure (usually a DNA–RNA heteroduplex) which cannot undergo ribosomal protein biosynthesis. Alternatively, the DNA–RNA heteroduplex can be a substrate for RNAse H-mediated degradation of the mRNA strand. This way, catalytic amounts of the ON can mediate the efficient cleavage of mRNA encoding a specific protein, which leads to effective (though reversible) and selective downregulation of the protein's activity. A third option for the biological action of oligonucleotide structures is the triggering of the RNA interference mechanism by double-stranded 'small interfering' RNA (siRNA, mechanism not shown) [4]. Alternatively, single-stranded oligonucleotides (anti-miRNA oligonucleotides, 'AMOs', 'antimiRs') can inhibit endogenous microRNA-mediated RNA interference by blocking the RNA strand in the involved protein–RNA complex (RISC) [5].

The capability of ONs to exert the aforementioned biological mechanisms via sequence-specific molecular recognition makes them highly attractive candidates for drug development. However, their pharmacokinetic properties are problematic and represent a significant hurdle for their therapeutic application. First, the high polarity of ONs, mainly caused by their oligoanionic phosphate diester backbone, severely hampers the penetration of biological barriers such as cellular membranes, thus leading to low cellular uptake. Second, unmodified ON structures are good substrates for nuclease-mediated degradation. Consequently, it is of vital importance to chemically modify ON structures in order to make them suitable drug candidates or chemical probes, e.g., for diagnostic purposes [6–7].

The relevance of the polyanionic phosphate diester-linked backbone to the overall function of nucleic acids has been discussed by Westheimer [8], Benner [9–10], and others. In spite of these considerations, many artificial internucleotide linkages were investigated in order to reduce the overall negative charge of the backbone and to enhance nuclease stability. One apparent approach to achieve these goals is the introduction of non-native electroneutral backbone linkages, with the nucleic acid mimic 'peptide nucleic acid' (PNA) [11–13] representing a striking example. Although the achiral PNA backbone is pronouncedly different from native nucleic acid structures, PNAs are capable of sequence-specific hybridization to native nucleic acids. However, their moderate water solubility and peptide-like folding properties [9] are hurdles for their biological application. As an alternative strategy, the (deoxy)ribose part of the backbone has been retained and only some of the internucleotide phosphate diesters have been selectively replaced by electroneutral motifs. Such artificial neutral linkages include, among others, sulfone [14], amide [15–22], triazole [23–27], phosphoramidate [28] and phosphate triester [29] moieties.

**Figure 2:**
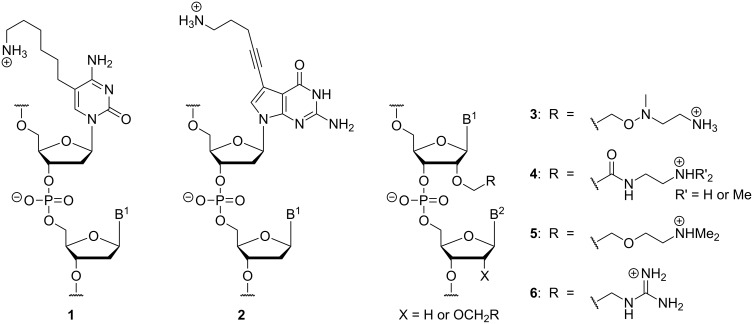
Selected examples **1**–**6** of nucleic acid modifications based on additionally attached positively charged moieties, but retaining an intact phosphate diester backbone (B^1^, B^2^ = nucleobases) [30–37].

Using a different approach, positive charges have been introduced into nucleic acid structures. Positively charged moieties were either employed (i) as additional charged structural motifs compensating for the negative charges in the backbone linkages or (ii) as replacements of the native negatively charged phosphate diester linkages. The first option has found considerable attention, with positively charged moieties attached to nucleobases or the ribose sugar. Some selected examples **1**–**6** of resulting nucleic acid structures are provided in Figure 2 [30–37]. Oligonucleotides of this type are at least partially zwitterionic, but overall densely charged. With respect to the aspired improvement of cellular uptake, fully cationic oligonucleotide analogues might also be attractive candidate structures, as indicated by the advantageous properties of cationic cell-penetrating peptides (CPPs) [38]. However, the design of modifications of type **1**–**6** precludes the preparation of fully cationic oligonucleotide analogues.

**Figure 3:**
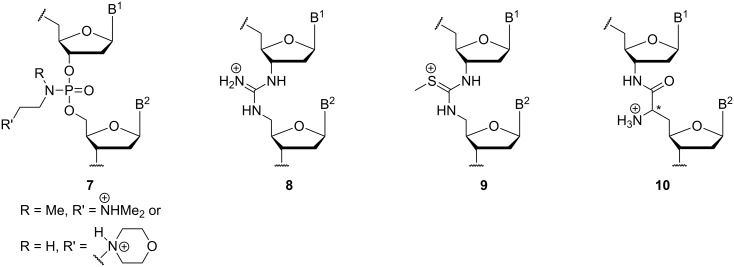
Oligonucleotide analogues with artificial cationic backbone linkages discussed in this review: aminoalkylated phosphoramidates **7** (and related systems, not shown), guanidinium-linked 'DNG' **8**, *S*-methylthiourea-linked oligomers **9**, and nucleosyl amino acid (NAA)-modified oligonucleotides **10** (B^1^, B^2^ = nucleobases).

This review focusses on the second aforementioned option to employ cationic motifs in oligonucleotide structures, i.e., as replacements of the native phosphate diester linkages [39]. In principle, this approach enables the preparation of partially or fully zwitterionic as well as cationic backbones. This strategy has been studied less frequently, with research on four artificial cationic linkages summarized in this review: aminoalkylated phosphoramidates (and related systems), guanidinium groups, *S*-methylthiourea motifs, and nucleosyl amino acid (NAA)-derived modifications. The synthesis and properties of the corresponding oligonucleotide analogues of types **7**–**10** (Figure 3) with cationic backbone linkages are described.

## Review

### Aminoalkyl phosphoramidate linkages and related systems

Pioneering work in the field has been reported by Letsinger and co-workers. In 1986, they introduced a deoxyadenosyl dinucleotide linked by an aminoethyl phosphoramidate moiety which is positively charged under acidic and neutral conditions [40]. Based on these results, they subsequently reported the synthesis of short, cationic DNA oligonucleotides with phosphoramidate linkages of type **7**, which were N-alkylated with substituents containing basic structural motifs [41].

**Scheme 1:**
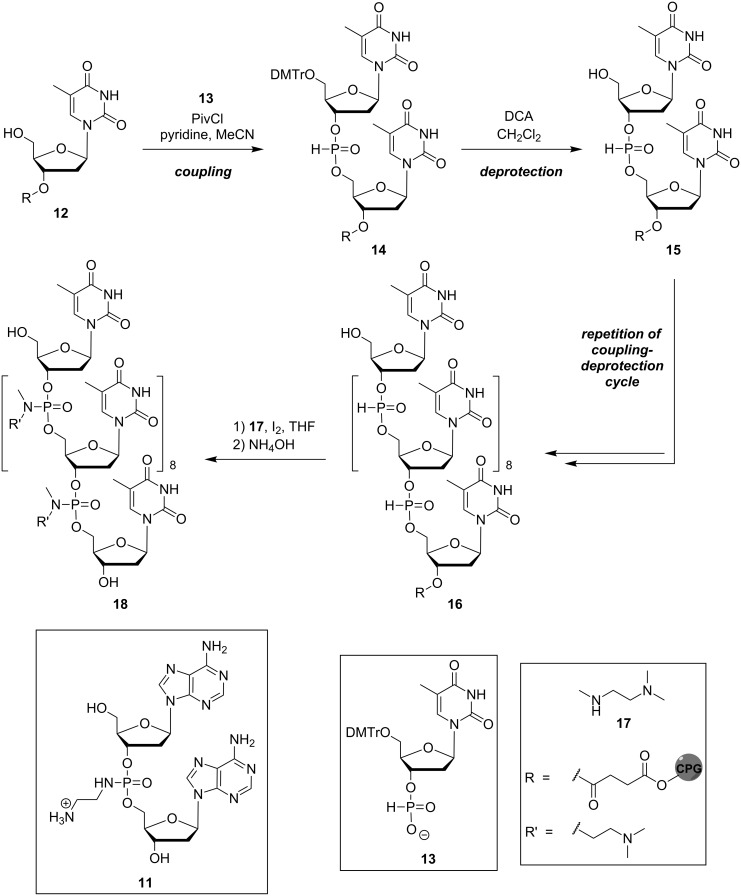
Structure of Letsinger's modified deoxyadenosyl dinucleotide **11** and synthesis of cationic oligonucleotide analogue **18** containing aminoalkyl phosphoramidate linkages. CPG = controlled pore glass (solid support).

The synthesis of the modified deoxyadenosyl dinucleotide **11** was achieved using solution-phase chemistry (reactions not shown, for structure of **11** see Scheme 1) [40]. Subsequently, the preparation of corresponding oligonucleotide analogues was performed on solid support using H-phosphonate chemistry (Scheme 1). Thus, solid phase-linked thymidine **12** was coupled with 5'-dimethoxytrityl-(DMTr)-protected thymidine 3'-H-phosphonate **13** to give dimeric H-phosphonate **14**, which was then acidically DMTr-deprotected to furnish **15**. After the desired number of such coupling-deprotection cycles, the phosphite-linked oligo-thymidine **16** was transformed in an oxidative amidation reaction [42] in the presence of iodine and *N*,*N*,*N'*-trimethylethylenediamine (**17**) to yield, after basic cleavage from the solid support, the envisioned aminoalkyl phosphoramidate-linked oligonucleotide **18**.

To study the hybridization properties of such cationic oligonucleotide analogues with native DNA and RNA, Letsinger and co-workers performed UV-monitored thermal denaturation experiments [40–41]. In the case of the modified deoxyadenosyl dimer **11**, hybridization with native RNA-T_Poly_ as well as with DNA-T_Poly_ strands was evident and the complex formed more stable than comparable complexes involving the native d(ApA) DNA reference. An increase of the measured *T*_m_ of ≈10 °C for complexes of the aminoethyl phosphoramidate-linked dinucleoside **11** with RNA and ≈25 °C for the according hybridization with DNA was observed [40]. In addition, the cationic dimer **11** was shown to bind more tightly to native RNA and DNA strands in the presence of magnesium chloride [40].

For the cationic T-oligomer **18**, Letsinger and co-workers reported a strongly reduced absorbance of a mixture of **18** with DNA-A_Poly_ in thermal melting studies, as compared to the non-hybridized, single-stranded oligonucleotides [41]. This indicated a successful complex formation with ordered base stacking of the positively charged oligonucleotide analogue and its native DNA counterstrand. When exposed to high ionic strength (1.0 M NaCl), the complex was shown to undergo a significant decrease in stability. This effect of high salt concentrations was inverse to the corresponding effect for native anionic DNA duplexes and obviously resulted from electrostatic shielding mediated by the salt ions, thus weakening the attraction of the oppositely charged backbones [41].

In order to elucidate the stability of aminoethyl phosphoramidate-linked oligonucleotides to nuclease-catalysed degradation, Letsinger and co-workers described the incubation of such oligomers, the deoxyadenosyl dimer **11** and DNA-T_Poly_ (as a reference) with snake venom phosphodiesterase and spleen phosphodiesterase, respectively [40–41]. In these assays, neither the modified dimer **11** nor oligonucleotides of type **7** (such as **18**) showed any degradation by either enzyme, while native DNA reference strands were rapidly cleaved.

Other groups have subsequently employed Letsinger's aminoalkyl phosphoramidate linkage (or variations thereof) in biochemical and biological studies on the properties of corresponding oligonucleotides. Weeks and co-workers have demonstrated that a triplex-forming antigene oligonucleotide modified with a variant of Letsinger's linkages can efficiently inhibit the expression of plasmid DNA injected into *Xenopus* oocytes [43]. The presence of the cationic backbone modification and a sufficiently long mismatch-free target DNA sequence were essential for this gene-silencing effect, thus indicating the relevance of enhanced nuclease stability and sequence-specific DNA binding. However, the gene-silencing effect could only be achieved if the modified oligonucleotide and the plasmid DNA were either mixed prior to cellular injection or if the oligonucleotide was injected first, pointing out a likely competition of the cationic antigene oligonucleotide with cellular histones for DNA binding [43].

Vasseur, Debart and co-workers have combined a variant of Letsinger's linkages with an α-configuration at the anomeric centers of antisense oligonucleotides [44–45]. They have found that such zwitterionic to fully cationic α-oligonucleotides bound to single-stranded DNA and RNA targets with high affinity, with duplex stabilization being proportional to the number of cationic modifications. It was also reported that these oligonucleotides showed retained base pairing fidelity, i.e., the *T*_m_ value was significantly reduced in the presence of a base mismatch. This specificity in binding suggested that such oligonucleotides should be promising sterically blocking antisense agents as their RNA targets were not digested by RNAse H. This anticipated bioactivity was confirmed in whole cell assays without the presence of transfection agents, suggesting that the altered charge pattern of the oligonucleotide backbone enabled its cellular self-delivery [44]. The same authors then also studied similar oligonucleotides with guanidinium groups as cationic moieties, which were obtained by postsynthetic guanidinylation of the congeners with amino-functionalized phosphoramidate linkages (reaction not shown) [46]. The presence of the guanidinium units furnished high hybridization affinities, in particular with single-stranded RNA targets, and also in triplex formation with double-stranded DNA, though the amino-functionalized analogues gave similar triplex stabilities. A fully cationic and fluorescently labelled guanidinylated oligonucleotide was subjected to comparative cellular uptake studies. Relative to its fluorescently labelled anionic phosphorothioate congener, it showed vastly enhanced cellular uptake. Fluorescence microscopy revealed a cytoplasmic localization of the oligonucleotide without accumulation in the nuclei. This indicated an endocytotic uptake mechanism with (at least partial) retention of the material in the endocytotic vesicles. No unspecific cytotoxic effect of the guanidinylated oligonucleotide was observed.

**Figure 4:**
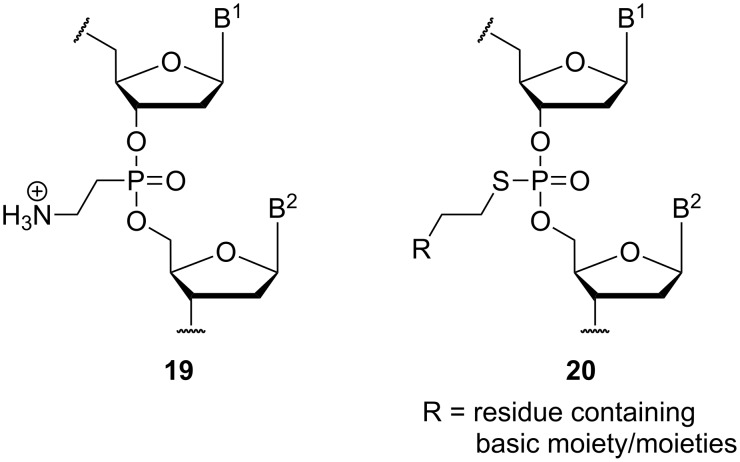
Artificial cationic backbone linkages **19** and **20** which are structurally related to aminoalkylated phosphoramidates of type **7** (B^1^, B^2^ = nucleobases).

Other types of oligonucleotides with aminoalkyl moieties as part of artificial internucleotide linkages have also been reported. With respect to their structural similarity to Letsinger's aminoalkyl phosphoramidate linkages, these variants are categorized as 'related systems' in this review. Fathi et al. have established the aminoethylphosphonate linkage **19** (i.e., a phosphonate analogue of amidate **7**) [47], and Rahman, Obika and co-workers have described cationic phosphorothioates of type **20** [48] (Figure 4).

The preparation of phosphonate linkage **19** was achieved in diastereomerically pure form, i.e., with defined configuration at the stereogenic phosphorus atom [47]. Corresponding *R*_P_-configured zwitterionic oligonucleotides formed duplexes with complementary DNA or RNA that were more stable than their respective native counterparts. The modified oligonucleotides showed pronounced nuclease and serum stability as well as significantly enhanced cellular uptake relative to their native congeners. As for the aforementioned phosphoramidates, fluorescence microscopy indicated a cytoplasmic localization of the tested zwitterionic oligonucleotide without significant accumulation in the nuclei, thus pointing to endocytotic uptake with retention of the compound in endocytotic vesicles (vide supra).

Cationically functionalized phosphorothioates of type **20** were also prepared as diastereomerically pure compounds with defined configuration at the stereogenic phosphorothioate unit [48]. A series of different residues (R in Figure 4) bearing one or two basic amino functionalities was introduced. The resulting 12-mer oligonucleotides with one cationic internucleotide linkage (all other linkages were phosphates) were tested for their ability to form duplexes with single-stranded DNA or RNA as well as triplexes with double-stranded DNA. The aminoalkylated *R*_P_-phosphorothioates showed an increased stability of DNA duplexes while the *S*_P_-isomers gave destabilized duplexes. Both the cationically functionalized *R*_P_- and *S*_P_-oligonucleotides displayed decreased affinity towards RNA, while triplex formation was enhanced for all tested *R*_P_ congeners. The aminoalkylation generally provided an increased nuclease stability, which was more pronounced for the *R*_P_ isomers.

### Deoxyribonucleic guanidines (DNG) with guanidinium linkages

In their design of cationic oligonucleotide analogues, Bruice et al. did not just attach a cationic moiety to the modified phosphate diester backbone, but they completely replaced it with a guanidinium linkage to give 'deoxyribonucleic guanidines (DNG)' of type **8** [49]. The guanidinium group was selected owing to its maintenance of a positive charge over a broad pH range and its ability to form both intermolecular electrostatic interactions and hydrogen bonds [50]. Letsinger's aminoalkyl phosphoramidate modification was stereogenic at the phosphorus atom, thus leading to complex mixtures of diastereomeric oligomers (with the exception of the aforementioned related systems, vide supra) as the stereoselective synthesis of stereogenic phosphate derivatives is challenging. Therefore, achiral artificial linkages such as guanidinium groups may be considered advantageous from a stereochemical perspective.

**Scheme 2:**
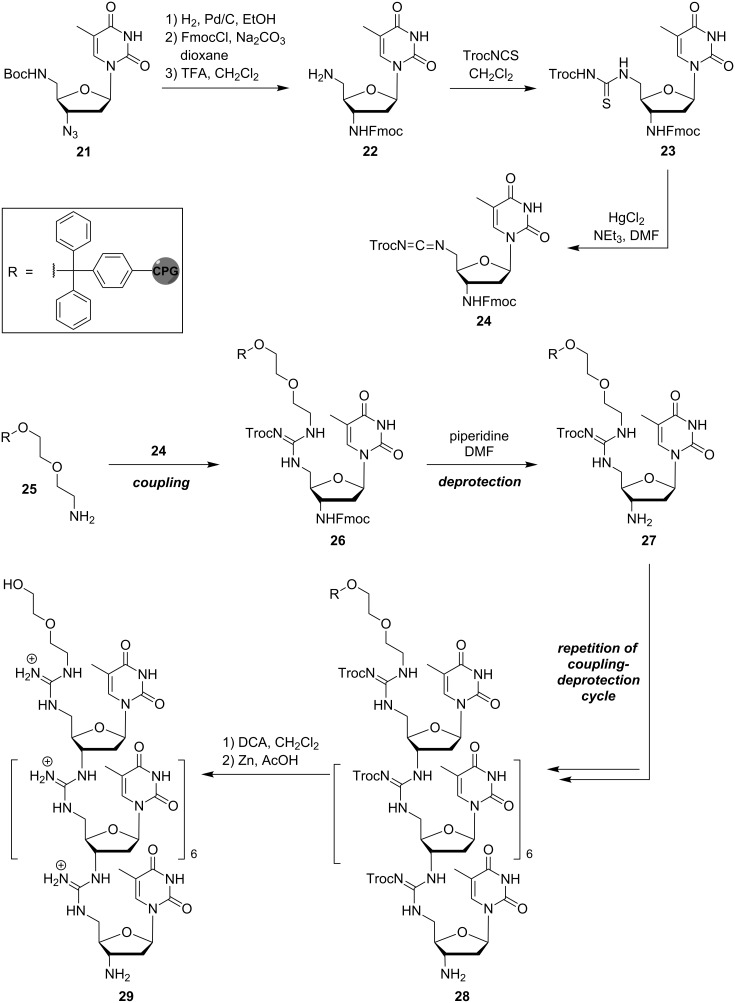
Bruice's synthesis of guanidinium-linked DNG oligomer **29** in the 5'→3' direction (Troc = 2,2,2-trichloroethyloxycarbonyl).

For the first synthesis of a pentameric thymidinyl DNG in 1996, Bruice and co-workers used an iterative solution-phase protocol (reactions not shown) [51]. This method was associated with some limitations, such as its moderate yields and the need for purification after each synthetic step. Subsequently, two different approaches for the solid phase-supported synthesis of DNG oligomers were introduced. They enabled chain elongation either in the 5'→3' [52] or 3'→5' [53] direction, respectively. Starting from protected 3',5'-dideoxy-5'-amino-3'-azidothymidine **21**, the 5'→3' route was based on the synthesis of the diamino intermediate **22** and thiourea monomer **23**, which was then converted into a reactive carbodiimide **24** and coupled to a terminal amino group of the solid phase **25** (Scheme 2). This coupling furnished solid phase-attached intermediate **26**, which was Fmoc-deprotected to the amine **27**. Iterative repetition of this coupling-deprotection cycle gave oligomer **28**, which was then acidically cleaved from the solid support and reductively Troc-deprotected to afford octameric thymidinyl DNG **29**.

**Scheme 3:**
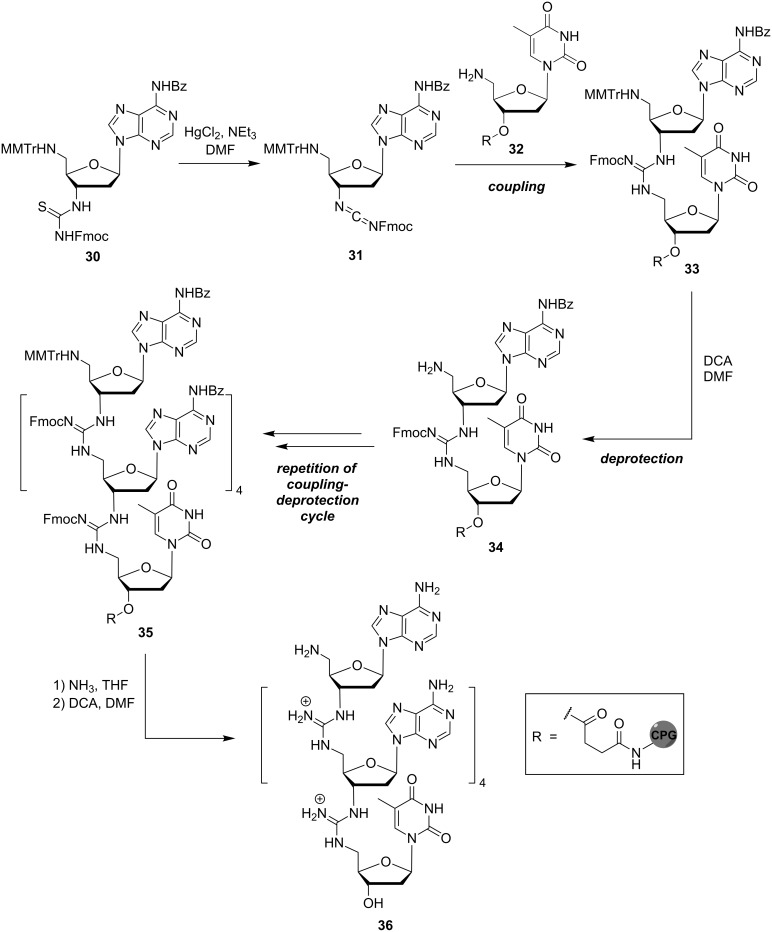
Bruice's synthesis of purine-containing guanidinium-linked DNG oligomer **36** in the 3'→5' direction (MMTr = monomethoxytrityl).

Based on this method, the solid phase-supported synthesis operating in the 3'→5' direction was later developed. As described by Bruice and co-workers, it was compatible with the cleavage conditions used in the solid phase-supported synthesis of native DNA and also allowed the introduction not only of pyrimidine, but also of purine bases into the oligonucleotide analogue [53]. The method was based on the activation of the 5′-monomethoxytrityl (MMTr)-protected 3'-thiourea monomer **30** to the corresponding carbodiimide **31** (Scheme 3). Using long-chain alkylamine controlled pore glass (CPG) loaded with 5′-amino-5′-deoxythymidine (**32**) as solid phase, the reaction cycle started with the guanidine-forming coupling of **31** and **32** to give **33**, followed by acidic cleavage of the MMTr protecting group to yield the free 5'-amine **34**. Subsequent iterative coupling–deprotection cycles resulted in the formation of the guanidinium-linked oligomer **35**. After basic guanidine and purine deprotection and concomitant cleavage from the solid support, final acidic deprotection furnished A_5_T oligonucleotide analogue **36**. In addition to these protocols, the solid phase-supported syntheses of DNG-DNA chimeras with partially zwitterionic backbone structures [54–55] as well as of further mixed DNG sequences [56–57] have been described (reactions not shown). It is also noteworthy that Bruice and co-workers succeeded in the preparation of corresponding guanidine-linked RNA analogues [58–59], though this is not within the main scope of this review.

Bruice et al. reported that oligonucleotide analogues containing the cationic DNG-modification bind to DNA with retention of base-pairing fidelity, furnishing thermally highly stable complexes with native complementary DNA and RNA counterstrands [51,60–64]. The increase in melting temperature for the DNG-DNA complex was reported to be around 15–25 °C per bp under nearly physiological conditions, dependent on the surrounding ionic strength. As shown by Job plot analysis, an oligo-thymidinyl DNG forms triple-stranded complexes in a 2:1 mixture with its native DNA counterstrand, i.e., the resulting triplex contains two DNG oligo-thymidylate analogues and one oligo-adenylate DNA strand [64]. The same binding stoichiometry was observed for an oligo-deoxyadenosyl DNG in complex with a native oligo-thymidylate DNA [53]. Overall, the obtained results suggest that adenosine- and thymidine-derived DNG oligomers support the formation of triplex structures, but that the DNG-DNA ratio within the complex is determined by the respective nucleobases. Remarkably, neither cytidinyl nor 7-deazaguanyl DNG oligomers furnish triplexes, but bind their complementary DNA counterstrand in a 1:1 ratio [65–66]. Furthermore, it was shown that an increase in ionic strength shields the oppositely charged backbones, thus destabilizing both DNG-DNA duplexes and triple-stranded DNG-DNA complexes, respectively. The triple-stranded DNG-DNA complex was less affected than its duplex congener though [51,60–61].

Regarding base-pairing fidelity, Bruice and co-workers have reported significantly reduced stabilities of DNG-DNA duplexes and triplexes, respectively, upon the insertion of base mismatches in the DNA counterstrand. Analyzing a 2:1 complex formed from two octameric thymidinyl DNG strands and one native DNA A_8_-mer, they concluded that base mismatches at either end of the DNA counterstrand sequence do not hamper hybridization as strongly as a single base mismatch in the center of the DNA strand. Two base mismatches in the center of the DNA counterstrand led to a complete loss of hybridization [64].

In addition to these thermal denaturation experiments, Bruice et al. also reported circular dichroism (CD) spectroscopic studies to obtain further information on the solution structures of DNG strands and their complexes with DNA. The corresponding analysis of the aforementioned triplex (DNG-T_8_)_2_/DNA-A_8_ indicated a usual B-DNA-derived triple helix structure, while the comparison of single-stranded DNG-T_8_ with native DNA-T_8_ furnished two very different CD spectra [64].

### *S*-Methylthiourea linkages

In addition to their work on DNG oligonucleotide analogues, Bruice et al. also reported the positively charged *S*-methylthiourea backbone modification as an artificial internucleotide linkage [67–69]. For oligomers containing this replacement of the backbone phosphate diesters, the term 'DNmts' was coined. Just like the guanidinium linkage in DNGs, the *S*-methylthiourea modification is not stereogenic and stable towards nuclease-mediated cleavage. Furthermore, it retains its positive charge independent of pH conditions.

**Scheme 4:**
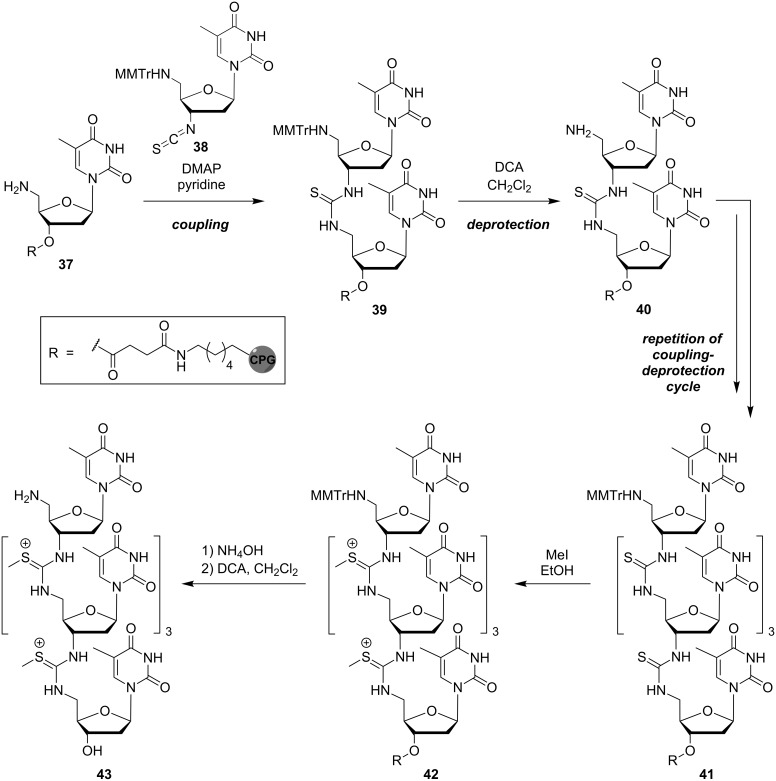
Bruice's synthesis of *S*-methylthiourea-linked DNmt oligomer **43**.

Bruice and co-workers initially reported a solution-phase synthesis that enabled the formation of pentameric thymidinyl DNmt in the 3'→5' direction (reactions not shown) [68]. They then introduced an automated solid phase-supported synthesis which was compatible with standard techniques of DNA synthesis (Scheme 4) [69]. A derivative of 5'-amino-5'-deoxythymidine attached to CPG (**37**) served as the solid phase. The construction of the oligomer, achieved in 3'→5' direction, was based on the coupling of 3'-isothiocyanate **38** with the 5'-amino group of **37** to give **39** and, after acidic MMTr cleavage, **40**. Iterative repetition of this coupling-deprotection cycle afforded thiourea-linked oligonucleotide analogue **41**. Subsequent reaction of the thiourea internucleotide linkages with methyl iodide furnished the protected *S*-methylthiourea-linked oligomer **42** and finally, after cleavage from the solid support and acidic deprotection, the envisioned DNmt oligomer **43**.

As for the pentameric DNG congener (vide supra), the DNmt-T_5_ oligonucleotide analogue was shown to bind more tightly to complementary DNA than DNA itself [68]. Under nearly physiological conditions with respect to pH and ionic strength, the *T*_m_ value for the DNmt-T_5_/DNA-A_Poly_ complex was reported to be above 80 °C whereas a comparable DNA–DNA duplex was only stable up to 13 °C. DNmt-T_5_ complexes with native RNA-A_Poly_ showed an even higher thermal stability. Job plot analysis revealed the formation of triple-stranded complexes between the DNmt pentamer and DNA-A_Poly_ or RNA-A_Poly_, respectively [68,70]. Similar to the results obtained for DNG-T_5_ (vide supra), a triplex with 2:1 stoichiometry (DNmt:DNA and DNmt:RNA, respectively) was confirmed.

Remarkably, Bruice et al. identified two different hyperchromic shifts for the DNmt-T_5_/DNA-A_Poly_ complex, but not for comparable DNmt-RNA aggregates when these mixtures were exposed to higher ionic strength, denoting the thermal denaturation of the (DNmt-T_5_)_2_/DNA-A_Poly_ triplex and, subsequently, the DNmt-DNA duplex. However, the corresponding melting temperatures were significantly lower than *T*_m_ values measured in aqueous solutions with physiological ionic strength. This indicates a pronounced destabilization of the DNmt-DNA complex with increasing ionic strength [70]. Comparable DNmt-RNA complexes were less destabilized under identical conditions.

Bruice and co-workers also performed further thermal denaturation studies to elucidate base-pairing fidelity of the pentameric thymidinyl DNmt. No increase in hyperchromicity was observed for combinations of DNmt-T_5_ with either DNA-G_Poly_, DNA-C_Poly_ or DNA-T_Poly_, over a temperature range from 5 to 93 °C, thus ruling out complex formation with these fully mismatched native DNA counterstrands. Furthermore, a pronounced drop in thermal stability of DNmt–DNA complexes containing 50% T–C mismatches and also for congeners containing 20% T–C mismatches was described [71].

In CD spectroscopic studies performed on the thymidinyl DNmt pentamer, Bruice et al. further confirmed the base-pairing specificity of oligonucleotides containing the artificial *S*-methylthiourea backbone linkage [70–71]. CD spectra of DNmt-T_5_ in complex with five different DNA oligonucleotides containing an increasing number of C mismatches showed significant changes dependent on the mismatch content. While the combination of DNmt-T_5_ with DNA-A_20_ resulted in a CD difference spectrum with distinct amplitude, the addition of DNA oligonucleotides with an increasing number of C mismatches led to continuous slackening of signals in the difference spectra, until those were almost flat for DNA oligonucleotides containing 50% C mismatches. Hence, this indicates that the ability of the DNmt pentamer to associate with a native DNA oligomer is dependent on Watson–Crick base pairing and is severely hampered by an increasing amount of base-pairing mismatches.

### Nucleosyl amino acid (NAA)-derived linkages

Both Letsinger's and Bruice's approaches for the introduction of positive charges into artificial backbone linkages have characteristic conformational features. Letsinger's aminoalkyl phosphoramidate modification and related systems involve a pronounced conformational flexibility of the moieties carrying the positively charged groups. Hence, it cannot be ruled out that interactions with the phosphate groups occur which would be less likely if the positively charged units were more rigidly fixed to the backbone. In contrast, both Bruice's DNG and DNmt oligonucleotide analogues are characterized by conformationally rigid internucleotide linkages. Apparently, an alternative strategy providing a positively charged backbone linkage with 'intermediate' conformational flexibility is missing.

**Figure 5:**
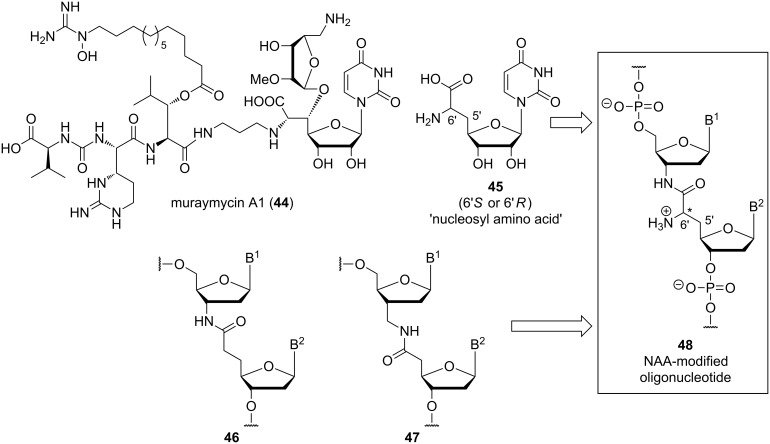
Structure of the natural product muraymycin A1 (**44**) and design concept of nucleosyl amino acid (NAA)-modified (partially) zwitterionic oligonucleotides of type **48** formally derived from structures **45**–**47** (B^1^, B^2^ = nucleobases).

These considerations have stimulated our design of a new artificial internucleotide linkage named 'nucleosyl amino acid (NAA)-modification' (Figure 5) [72–74]. In principle, the NAA-modification is inspired by 'high-carbon' nucleoside structures (i.e., nucleosides having more than five carbon atoms in the sugar unit) found in naturally occurring nucleoside antibiotics [75–77]. In muraymycin- and caprazamycin-type nucleoside antibiotics, among others, such 'high-carbon' nucleosides are uridine-derived amino acid structures ('glycyluridine', GlyU) [78–80], which are aminoribosylated at the 5'-hydroxy group. As part of our ongoing research program on muraymycin nucleoside antibiotics (e.g., muraymycin A1 (**44**)) and their analogues [81–88], we have reported the synthesis of simplified (i.e., 5'-defunctionalized) GlyU derivatives of type **45** (Figure 5) [86–88]. The formal amalgamation of this 'nucleosyl amino acid (NAA)' structure **45** with previously reported amide internucleotide linkages of types **46** and **47** [15–22] furnished the structure of an 'NAA-modified oligonucleotide' **48** (Figure 5). The 6'-amino group of the NAA-modification is positively charged at physiological pH values, thus providing a (partially) zwitterionic backbone structure if some phosphate diester units are replaced with the NAA-modification. In the NAA-modification, several rotatable bonds are combined with the rigid amide group, and it is therefore expected to represent an example of the aforementioned positively charged backbone linkage with 'intermediate' conformational flexibility (vide supra).

**Scheme 5:**
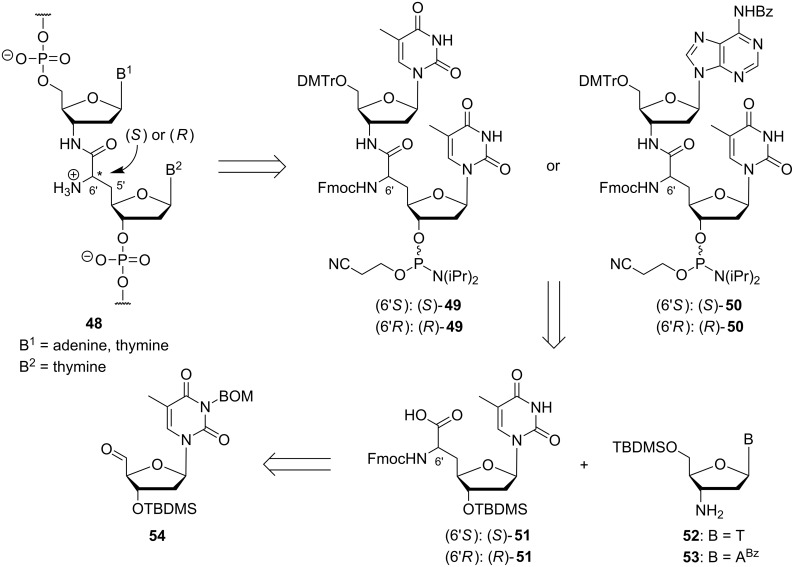
Retrosynthetic summary of Ducho's synthesis of partially zwitterionic NAA-modified oligonucleotides **48** (BOM = benzyloxymethyl).

We have reported that partially zwitterionic NAA-modified DNA oligonucleotides can be obtained by standard solid phase-supported automated DNA synthesis if 'dimeric' phosphoramidite building blocks **49** and **50** are employed (Scheme 5) [72–73]. For the synthesis of 'dimeric' phosphoramidites **49** and **50**, protected thymidinyl amino acids (*S*)-**51** or (*R*)-**51** were coupled with protected 3'-amino-3'-deoxythymidine **52** or protected 3'-amino-2',3'-dideoxyadenosine **53** [73,89], respectively. Thymidinyl amino acids **51** were obtained from 3'-*O*-silylated thymidine-5'-aldehyde **54** via a previously established route using Wittig–Horner olefination and catalytic asymmetric hydrogenation as key steps (reactions not shown) [86–87,90–92].

Using 'dimeric' building blocks (*S*)-**49**, (*R*)-**49**, (*S*)-**50**, and (*R*)-**50** (Scheme 5), automated DNA synthesis under standard conditions enabled the preparation of partially zwitterionic NAA-modified oligonucleotides with defined configuration at the 6'-position, i.e., with control over the spatial orientation of the positive charge [72–73]. Thus, the NAA-modification was placed in T–T ('TxT', with x representing the NAA-linkage) and A–T segments ('AxT') of the oligonucleotide sequence, respectively. Further variation of the 3'-aminonucleoside component (**52** and **53** in Scheme 5) should potentially also allow the introduction of the NAA-modification at C–T and G–T sites within a given sequence.

So far, 24 different oligonucleotides with one to four TxT NAA-modifications at various positions [72] as well as two oligonucleotides with two AxT NAA-modifications [73] have been reported. The properties of the TxT-containing congeners have been studied in detail [72]. Thermal denaturation experiments showed that the TxT NAA-modified DNA oligonucleotides formed duplexes with complementary native DNA or RNA counterstrands, but with moderate destabilization relative to unmodified native duplexes, in particular for DNA–RNA hybrids. The fidelity of base pairing was studied using native DNA counterstrands containing a single base mismatch. Furthermore, structures of the duplexes were investigated by CD spectroscopy. The following properties of TxT NAA-modified DNA oligonucleotides were reported [72]: (i) they formed reasonably stable duplexes with complementary counterstrands, in particular with native DNA; (ii) the influence of the spatial orientation of the positive charge, i.e., of the configuration at the 6'-position, was moderate, with a tendency that (6'*R*)-configured linkages furnished slightly more stable duplexes; (iii) the modified oligonucleotides showed no impairment of mismatch discrimination, i.e., single base mismatches led to a significant drop in duplex stability; (iv) the formed duplexes were devoid of significant structural distortion, i.e., their CD spectra indicated B-type helices for DNA–DNA duplexes and A-type helices for DNA–RNA duplexes. Overall, these results demonstrated that typical chemical properties of nucleic acids are retained in partially zwitterionic NAA-modified DNA oligonucleotides. However, corresponding studies on NAA-modified DNA oligonucleotides with a fully zwitterionic backbone have not been conducted yet.

**Scheme 6:**
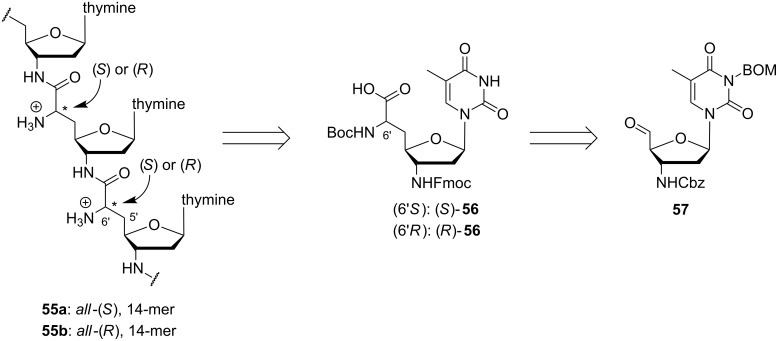
Retrosynthetic summary of Ducho's and Grossmann's synthesis of fully cationic NAA-modified oligonucleotides **55a** and **55b**.

With respect to the aforementioned favourable properties of zwitterionic NAA-modified oligonucleotides, the obvious aim was to synthesize fully cationic oligomers, i.e., oligonucleotide analogues with the cationic NAA-modification as their sole internucleotide linkage. The phosphoramidite-based synthetic strategy depicted in Scheme 5 was not suitable to reach this goal as it furnishes phosphate diester linkages at least at every second position within a given sequence. Therefore, a different synthetic route was developed (Scheme 6) [74]. The envisioned fully cationic thymidine-derived oligomers **55a** (*all*-(*S*)-configured at the 6'-positions) and **55b** (*all*-(*R*)-configured at the 6'-positions) were assembled by manual Fmoc-based solid phase-supported peptide synthesis using the monomeric 3'-amino-nucleosyl amino acids (*S*)-**56** and (*R*)-**56**, respectively, as building blocks. The synthesis of thymidinyl amino acids **56** was again started from a corresponding 5'-aldehyde **57** using Wittig–Horner olefination and catalytic asymmetric hydrogenation as key steps (reactions not shown) [74].

The properties of fully cationic oligonucleotide analogues **55a** and **55b** were studied in detail [74]. Thermal denaturation experiments demonstrated a strong hybridization of both thymidinyl oligomers with native complementary A_14_ DNA, with *T*_m_ values being 9 and 17 °C higher, respectively, than the *T*_m_ value of an unmodified T_14_–A_14_ DNA reference duplex. As anticipated based on Letsinger's and Bruice' work (vide supra), the *T*_m_ value of the **55**–DNA complex decreased with increasing ionic strength. Studies on base-pairing fidelity gave the remarkable result that both **55a** and **55b** were largely insensitive to the presence of a single base mismatch in the counterstrand, thus indicating that electrostatic attraction overruled Watson–Crick base-pairing specificity in these cases. CD spectroscopy indicated that both **55a** and **55b** formed double-helical duplex structures with complementary DNA, apparently with slight distortions in case of the **55b**–DNA duplex.

The hampered base-pairing fidelity of **55a** and **55b** raised the question if the hybridization of these oligocations with oligoanionic DNA was dependent on Watson–Crick base-pairing at all or if it was mainly mediated by electrostatic attraction. Thermal denaturation studies of mixtures of **55a** or **55b**, respectively, with a fully mismatched DNA counterstrand (G_6_TTG_6_) showed a pronounced hyperchromicity upon heating in both cases, but also indicated that no transition between two defined states occurred [74]. It was derived from these results that **55a** and **55b** probably formed less defined, unspecific aggregates with the fully mismatched counterstrand, which then disassembled at elevated temperatures. This hypothesis was further supported by CD-spectroscopic studies. The overall conclusion was that the formation of defined double-helical duplex structures of **55a** and **55b** with DNA was mainly steered by Watson–Crick base-pairing, but that unspecific electrostatic attraction also contributed to the hybridization of the strands.

## Conclusion

In summary, this review provides an overview of four different approaches to introduce cationic backbone linkages as replacements of the phosphate diester units into oligonucleotide structures: i) aminoalkylated phosphoramidates and related systems; ii) guanidinium groups; iii) *S*-methylthiourea motifs and iv) nucleosyl amino acid (NAA)-derived modifications. All of these artificial internucleotide linkages are accessible by means of chemical synthesis, which is either based on the application of H-phosphonate (for i) or phosphoramidite-based (for iv) DNA synthesis, or on a massively modified version of DNA synthesis (for ii and iii), or on solid phase-supported peptide synthesis (for iv).

Studies on the properties of resulting oligomers are not fully conclusive yet. Some data, for instance on base-pairing fidelity, are missing for Letsinger's originally reported aminoalkylated phosphoramidates, while subsequently reported variants thereof and related systems have been studied in more detail. Thus, both retained base-pairing fidelity and improved cellular uptake have been reported for some oligonucleotides with structural similarity to Letsinger's first-generation aminoalkylated phosphoramidates. Bruice's guanidinium- and *S*-methylthiourea-linked systems have a pronounced tendency to form triple-helical structures with native nucleic acids, which makes a direct comparison with the other approaches difficult. Bruice's data suggest retained base-pairing fidelity for fully cationic oligomers, which is in remarkable contrast to our results obtained for NAA-modified oligonucleotides. The latter showed excellent base-pairing fidelity in the case of partially zwitterionic backbones, but insensitivity to single base mismatches for the hybridization of fully cationic oligomers with native DNA. Recently reported results on such fully cationic NAA oligomers [74] indicate that in addition to Watson–Crick base-pairing, unspecific electrostatic attraction also plays a role in the hybridization process. Overall, one must state that the interplay of the structural and conformational properties of cationic internucleotide linkages and the physicochemical behaviour of corresponding oligomers in their binding to anionic nucleic acids is only scarcely understood and will require further research efforts.

Studies on the biological properties of (partially) zwitterionic and cationic oligonucleotide analogues in cellular systems, in particular with respect to their cellular uptake, are currently only available for some aminoalkylated phosphoramidate-linked oligonucleotides and a related phosphonate analogue. The anticipated vast improvement of cellular uptake due to the presence of the cationic internucleotide linkages was proven for these systems, even though they displayed hampered endosomal release. On the other hand, our results on NAA-derived cationic oligomers suggest that, as a paradigm for the design of cationic oligonucleotide analogues for biological applications, one should potentially be cautious with respect to the number of positive charges in the backbone: base-pairing fidelity might be hampered, dependent on the structure of the artificial internucleotide linkage. It will therefore also be of significant relevance to further investigate the influence of the charge pattern in the backbone on the oligonucleotides' cellular uptake. The stage is set to perform such studies, which will further advance the development of cationically linked oligonucleotide analogues for potential applications as drug candidates, diagnostic agents or chemical tool compounds.
